# Latent Profiles of Professional Identity Formation, Professionalism, Leadership, Resilience, and Well-being among Medical Students

**DOI:** 10.1007/s40670-026-02820-6

**Published:** 2026-07-16

**Authors:** Dick Nsimbe, Anne Hickey, Denis W. Harkin, Abdelsalam Bensaaud, Áine Ryan, Frank Doyle

**Affiliations:** 1 Department of Health Psychology, School of Population Health, RCSI University of Medicine and Health Sciences, 123 St Stephen’s Green, Dublin 2, D02 YN77 Dublin 2, Dublin, Ireland; 2 Centre for Professionalism in Medicine and Health Sciences, RCSI University of Medicine and Health Sciences, Dublin, Ireland

**Keywords:** Latent profile analysis, Professional identity formation, Professionalism, Leadership, Resilience and well-being

## Abstract

**Introduction:**

Professional Identity Formation (PIF) is central to medical education, reflecting how students internalize the values, behaviours, and roles of the profession. While interest in PIF is increasing, limited work has explored how professional identity, professionalism, leadership, resilience, and well-being co-occur within subgroups. Most studies use variable-centred approaches, overlooking subgroup differences. This study aimed to use Latent Profile Analysis (LPA) to identify distinct sub-groups of medical students based on these constructs, exploring implications for future targeted educational support.

**Methods:**

A cross-sectional survey was conducted among 679 national and international medical students at the RCSI University of Medicine and Health Sciences, Ireland, using 76 items drawn from four validated scales measuring PIF, Professionalism, Leadership, Resilience, and Well-being. LPA was applied to uncover unobserved subgroups, and multinomial logistic regression estimated the associations between covariates and profile membership.

**Results:**

LPA identified a three-profile solution as optimal: *Conscientious Followers* (*n* = 322, 47%), *Development Group* (*n* = 196, 29%), and *Resilient Leaders* (*n* = 161, 24%) based on distinct patterns of scores across different constructs. *Conscientious Followers* was characterized by higher scores on perceptions of professional identity, psychological well-being, and professionalism, but lower scores on resilience and leadership. The *Development Group* demonstrated the lowest scores across leadership, professionalism, perceptions of professional identity, and psychological well-being, while showing moderate levels of resilience. *Resilient Leaders* displayed higher resilience and leadership scores, moderate perceptions of professional identity and professionalism, and lower psychological well-being. Multinomial logistic regression results showed that students’ region of origin and year of study were associated with group membership.

**Conclusion:**

Medical students demonstrate distinct latent profiles across professional identity formation, professionalism, leadership, resilience, and well-being. These findings highlight the need for tailored educational support that addresses the distinct developmental needs of each subgroup, alongside developmentally appropriate interventions to foster students’ evolving professional identities and overall well-being throughout medical training.

**Supplementary Information:**

The online version contains supplementary material available at https://doi.org/10.1007/s40670-026-02820-6.

## Background

Professional Identity Formation (PIF) is increasingly recognized as a central developmental task in medical education, reflecting the internalization of the values, norms, and responsibilities of the profession [1–3]. As students’ progress through medical school, they are expected to evolve not only in knowledge and skill but also in respect to professional identity, from layperson to physician [4]. This transformation is shaped by curricular, interpersonal, and institutional influences [2]. Closely linked to PIF are the constructs of professionalism, leadership, resilience and well-being, each essential for effective, ethical, and sustainable medical practice [5]. Professionalism encompasses values, professional behaviours, and attitudes expected from healthcare professionals to maintain public trust and ensure patient safety [3, 6]; leadership is the development of skills and behaviours that enable graduates to coordinate teams, make informed decisions, and positively influence patient care [7]; and resilience is the ability to adapt well in the face of adversity, trauma, tragedy, threats, and significant sources of stress, and is critical for maintaining mental health in medical education [8, 9]; and well-being reflects positive psychological functioning and flourishing, including engagement, perseverance, optimism, connectedness, and happiness, which support students’ ability to thrive during medical training [10].

Previous research has shown that professionalism, leadership, and resilience are important contributors to the professional development and identity formation of medical students [5, 11]. Although previous studies have traditionally examined these constructs in isolation [3, 7, 9], emerging evidence suggests they are conceptually linked and tend to co-occur within individuals [5, 12, 13]. Ryan et al. [5, 11] provided empirical evidence that these constructs are interrelated and contribute collectively to professional development and identity formation. Although well-being is conceptually viewed as an outcome arising from multiple interacting personal and educational factors, rather than an inherent component of professional identity formation itself, the original PIF, Professionalism, Leadership, Resilience (PILLAR) methodology incorporated well-being alongside resilience as an important indicator of students’ psychosocial functioning and capacity to flourish during medical training. However, recent longitudinal evidence suggests that these constructs may follow different developmental trajectories over the course of medical training, with professional identity formation increasing progressively while professionalism, leadership, and resilience remain relatively stable [14]. Together, these findings highlight the complexity of professional development and suggest that, although these competencies are related, they may manifest differently across learners. This underscores the need to understand how they co-occur within individual students.

According to Cruess et al.’s conceptualisation of professional identity formation, PIF involves the internalization of professional values and roles through socialization and experiential learning [12]. While traditional variable-centered research has clarified how individual competencies such as professionalism, leadership, and resilience and well-being contribute to PIF, it assumes a homogeneous learner population and may overlook unobserved patterns. Applying a person-centred approach such as Latent Profile Analysis (LPA) allows for the identification of latent subgroups of students whose profiles across these competencies may reflect distinct pathways of professional identity formation. For medical educators, this provides an opportunity to better understand variation in learners’ developmental experiences and support needs rather than assuming a uniform pattern of professional development across all students. This approach provides novel theoretical insights into the heterogeneous nature of PIF, extending our understanding of how multiple interrelated constructs integrate to shape professional development.

Despite growing attention to professionalism, leadership, and resilience in shaping medical students’ professional identity [3, 5, 15], much existing research relies on variable-centred methods. Techniques like regression, path analysis and structural equation modelling (SEM) effectively highlight associations between constructs, but they assume participants come from a single, homogeneous population thereby overlooking important individual-level variations [16–18]. For instance, two students might have similar professionalism scores yet differ significantly in leadership or resilience, differences which variable-centred techniques often overlook. Given the individualized and dynamic nature of PIF (1, 2), person-centred approaches like LPA provide a deeper insight into how these competencies cluster within individuals.

Furthermore, LPA allows the identification of unobserved subgroups within a population based on shared patterns across multiple indicators [16–18]. These approaches acknowledge that development varies across individuals, with unique profiles that may require tailored educational strategies and support. In medical education, where learners follow varied trajectories and bring diverse life experiences [19, 20], capturing this heterogeneity is crucial. Prior studies have successfully applied LPA to identify subgroups in professionalism, resilience, leadership styles, and stress-coping patterns. For instance, Jiang et al. [21] identified three professionalism “calling” profiles among nursing students, differentiated by demographic and experiential factors, highlighting the need for tailored professional development. Song et al. [22] classified medical students into low, moderate, and high resilience groups, showing differences in mental health and coping patterns, with female students with less-educated parents being at greater risk for low resilience. Zhang et al. [23] found four self-leadership profiles among general practice students, which predicted distinct coping styles, suggesting targeted interventions could enhance self-leadership and stress management. Similarly, Sun et al. [24] identified four stress-coping profiles among nursing students during the COVID-19 pandemic, with differences in coping strategies and professional identity. Collectively, these studies demonstrated the potential of the LPA method to uncover unobserved subgroups among learners in individual areas related to PIF. However, no study to date has applied LPA to examine PIF subgroups and its related constructs of professionalism, leadership, resilience, and well-being in one analysis.

Integrating professionalism, leadership, resilience, and well-being into a single analysis provides a more realistic approach to understanding how medical students experience professional development. These competencies do not develop in isolation, they interact and co-develop with one another and reinforce each other throughout medical training [5, 11–13]. Identifying how these constructs co-occur within individuals can enable educators to detect students who may be at greater risk, for instance, those exhibiting low resilience or limited self-perceived leadership skills as well as those who demonstrate strengths that could be further nurtured. This knowledge has the potential to inform more tailored curricular design, mentoring approaches, and student well-being interventions. Furthermore, LPA may help identify students who exhibit low professionalism, thereby enabling targeted remediation to reduce the risk of unprofessional behaviours [25]. Accordingly, this study sought to answer the following research question: Can distinct latent profiles of medical students be identified based on PIF, professionalism, leadership, resilience, and their relationship to well-being, and what demographic factors are associated with membership in these profiles? To address this question, we applied a person-centred latent profile approach to identify naturally occurring subgroups of medical students based on PIF, professionalism, leadership, resilience, and well-being, thereby advancing the theoretical understanding of professional development and professional identity formation in medical education.

## Methods

This study was reported in accordance with the Strengthening the Reporting of Observational Studies in Epidemiology (STROBE) guidelines [26]. The completed STROBE checklist is provided in the Supplementary Material. Ethical approval for the study was granted by the RCSI Research Ethics Committee (REC202005016), and all participants provided informed consent prior to participation.

### Study Design and Participants

This study employed a cross-sectional design, using data collected from medical students at the RCSI University of Medicine and Health Sciences, Dublin Campus. Participants included students enrolled in either the Direct Entry Medicine (DEM) 5-year or Graduate Entry Medicine (GEM) 4-year programmes. The GEM programme compresses the first three years of the DEM programme into two years and then both groups merge for the final two years of the programme. For the purposes of this analysis, both groups were treated as a single cohort to explore overall patterns across the student population. RCSI delivers a standard medical curriculum which integrates foundational biomedical sciences with clinical applications and includes learning activities to encourage professional development. All students completed a set of validated instruments comprising 76 items designed to assess various aspects of professional development [5, 11]. These assessments were embedded within the curriculum. While completion of the assessments was required for academic purposes, participation in the research context was voluntary and based on fully informed consent, which was obtained at the time of data collection.

### Setting

The assessments were administered online to medical students enrolled at RCSI University of Medicine and Health Sciences, Dublin.

### Study Size

The study comprised of 679 medical students enrolled in either the DEM or GEM programmes between 2019 and 2024. Full details on the methods have been reported previously [11]. The instruments were selected following the methodology of the original PILLAR study [11], which assessed Professional Identity Formation, professionalism, leadership, resilience, and well-being as complementary dimensions of medical students’ professional. A thorough literature review was conducted to identify potential measures for each construct, after which an expert panel of educators, psychologists, and clinicians agreed on the most suitable scale for each domain, considering factors such as content relevance, psychometric quality, suitability for the population, reliability, and free accessibility, in line with the original PILLAR approach.

### Variables

The PILLAR assessment included well-established and standardized instruments designed to evaluate PIF, professionalism, leadership, resilience, and well-being [11]. Consistent with the original PILLAR methodology, well-being was included alongside the other constructs because positive psychological functioning is considered an important component of students’ professional development and capacity to thrive during medical training. Although conceptually distinct from professional identity formation, well-being provides an important indicator of how students experience and adapt to the demands of medical training. While resilience reflects the ability to recover from stress and adversity, well-being captures broader dimensions of flourishing, including engagement, perseverance, optimism, connectedness, and happiness.

### Professional Identity Formation (PIF)

Professional identity was assessed using the 9-item Professional Self-Identity Questionnaire (PSIQ) [27], which captured participants’ confidence in undertaking core professional activities within healthcare. The instrument covered domains in communication, teamwork, patient assessment, ethical practice, and reflective learning. Each item was rated on a scale ranging from 0 (first-day medical student) to 6 (newly qualified doctor). Aggregated scores were derived by averaging responses, with higher values indicating stronger self-perceived professional identity. The scale demonstrated excellent internal consistency in this sample (Cronbach’s α = 0.94).

### Professionalism

Professionalism was assessed using a revised 27-item scale that measures individuals’ perceptions of and involvement in unprofessional behaviours [28]. For the current study, 25 items were retained after removing two items related to inappropriate dressing [11]. Participants were asked to indicate whether they had observed or engaged in each behaviour and whether they considered it unprofessional. In line with our previous studies, which focused on analysing perceptions of professionalism to streamline the analysis, this study maintains the same conceptual focus to ensure consistency (α = 0.89) [5, 29].

### Leadership

Leadership competencies were evaluated using items adapted from the Medical Leadership Competency Framework (MLCF) [30]. The tool assessed attributes related to self-awareness and teamwork via 15 items, each rated on a 3-point scale: 0 (“very little/none of the time”), 1 (“some of the time”), or 2 (“a lot of the time”). Higher average scores indicated greater perceived leadership ability. Reliability for this measure in the current sample was acceptable (α = 0.82).

### Resilience

The Brief Resilience Scale (BRS) [31] was used to assess resilience. This is a 6-item measure assessing the ability to recover from stress, using a 5-point Likert scale from “strongly disagree” to “strongly agree.” Higher average scores reflect stronger resilience (α = 0.74).

### Well-Being

Student well-being was measured using the EPOCH Measure of Adolescent Well-being [10]. This 20-item tool captures five dimensions of well-being, Engagement, Perseverance, Optimism, Connectedness, and Happiness. Each domain is scored based on the mean of four items rated on a 1 (“never”) to 5 (“always”) scale. Internal consistency for the scale was good (α = 0.80). For parsimony in this analysis, the EPOCH measure was considered as a total without delving into individual dimensions. Unlike resilience, which focuses primarily on recovery from adversity, the EPOCH measure captures broader aspects of positive psychological functioning and flourishing, representing an important outcome of students’ overall educational and professional experiences.

### Data Sources

Data for this study were obtained through the RCSI Quality Enhancement Office (QEO), which served as the independent data controller to ensure the confidentiality and security of participant information. The Professional Identity Formation, Professionalism, Leadership, and Resilience (PILLAR) assessment was administered via a secure online survey platform to protect the integrity and privacy of responses. Prior to survey distribution, study investigators delivered brief presentations to each cohort of medical students, outlining the study’s objectives and relevance. Following these introductions, the QEO disseminated individualized survey links to all eligible students through institutional email, integrated within their respective academic modules. To optimize response rates, up to two reminder emails were issued weekly for a period 3 weeks. Upon accessing the link, participants were presented with an electronic participant information sheet and informed consent form. Only those who provided informed consent were included in the final sample. To uphold confidentiality, the QEO implemented a two-stage de-identification process, initial pseudonymization to enable accurate linkage and credit assignment, followed by complete anonymization through the removal of all identifiable data prior to transfer to the research team. This protocol ensured both data integrity and participant anonymity.

### Statistical Analysis

All analyses were conducted using R software (version 4.5.0). A three-step analysis approach was employed. First, descriptive statistics were computed to summarize participants’ demographic characteristics. Second, a Latent Profile Analysis (LPA) was conducted to classify students into unobserved subgroups based on their scores in PIF, professionalism, leadership, resilience, and well-being. Standardization was applied to facilitate meaningful comparison across constructs measured on different scales. Each variable was transformed into a z-score, centering at a mean of zero and scaling by standard deviation, this allowed a clear interpretation of relative strengths and weaknesses. It was assumed that each student belonged to one of a finite number of latent profiles, the number and size of which were unknown a priori [32]. The analysis began with a one-class solution, after which additional profiles were incrementally added. Models ranging from one to 9 classes were estimated to determine the optimal number of latent profiles. The most suitable model was selected based on the fit indices, including loglikelihood test, Akaike Information Criterion (AIC), Bayesian Information Criterion (BIC), Consistent AIC (CAIC), Sample-Size Adjusted BIC (SABIC), and the Bootstrap Likelihood Ratio Test (BLRT). Lower values of AIC, BIC, CAIC, and SABIC indicated better model fit [33–35]. In addition, we assessed the quality of the classification using entropy. Scores ≥ 0.8 indicated a clear separation between the different profiles and greater classification accuracy. BLRT was employed to compare neighbouring profiles and to evaluate their improvement in fit. A statistically significant P-value suggests that the model with K classes is a significant improvement over the model with K – 1 classes [36]. In addition to statistical fit, model selection also considered practical factor of minimum profile sample size (n_min) and interpretability. Several studies have indicated that profiles with small sample sizes tend to be unstable and difficult to replicate [17, 34, 37–40]. Based on this literature, we considered that profiles comprising fewer than 10% of the total sample should be excluded during final model selection. Once profiles were identified, they were named to accurately indicate their features and differentiate them from other profiles. Third, a multinomial logistic regression was used to determine factors associated with profile membership.

## Results

Table 1 presents the demographic and background characteristics of the study participants (*N* = 679). The majority (70%) were enrolled in the Direct Entry Medicine program. Participants ranged in age from 18 to 40 years, with a mean age of 22.5 years (SD = 3.6) and a median age of 22 years. The majority identified as female (58%). The largest proportion were from the Middle East (32%), followed by Irish (27%) and North American (21%).

**Table 1:** Participant demographics and background characteristics

Variables	Statistic/Category	(*N* = 679)
Program	DEM	475 (70%)
	GEM	204 (30%)
Age	Min– Max	18–40
	Mean (SD)	22.5 (3.6)
	Median (Interquartile Range)	22 (20–24)
Gender	Female	394 (58%)
	Male	285 (42%)
Nationality	Irish	182 (27%)
	Middle Eastern	220 (32%)
	North American	139 (21%)
	Other (incl. EU, Asia, Africa)	138 (20%)

Abbreviations:SD, standard deviation; DEM, Direct Entry Medicine; GEM, Graduate Entry Medicine

Table 2 presents the comparative model fit indices for latent profiles among medical students. Based on these fit statistics, the three-profile (class) model was selected as the optimal solution. It demonstrated substantial improvement over the two-profile solution across multiple indices, including the loglikelihood test, AIC, BIC, CAIC, and SABIC. The entropy value of 0.87 indicated high classification accuracy, exceeding the conventional threshold of 0.80. BLRT was statistically significant (*p* = 0.01), further supporting the superiority of the three-profile solution. The minimum profile sample size (n_min) was 24%, which was considered reasonable and valuable in comparison to the smaller groupings observed in four or more profile solutions (e.g., 6%, 5%, 4%, 3%). Although models with additional classes yielded marginal improvements in statistical fit, these came at the cost of interpretability and an increased risk of overfitting. As a result, the three-profile model represents the most appropriate balance between model fit, theoretical interpretability, and parsimony.

**Table 2:** Comparative model fit indices for latent profiles among medical students

Model	LogLik	AIC	BIC	CAIC	SABIC	Entropy	n_min	n_max	BLRT (*p*)
1 Profile	−6857.37	13746.74	13816.87	13832.87	13766.08	1.00	1.00	1.00	*NA*
2 Profile	−6626.68	13303.36	13412.95	13437.95	13333.58	0.84	0.26	0.74	461.38 (0.01)
3 Profile	−6555.90	13179.81	13328.84	13362.84	13220.91	0.87	0.24	0.47	141.56 (0.01)
4 Profile	−6378.93	12843.86	13032.35	13075.35	12895.84	0.87	0.06	0.46	353.95 (0.01)
5 Profile	−6303.41	12710.83	12938.77	12990.77	12773.69	0.84	0.06	0.43	151.03 (0.01)
6 Profile	−6255.68	12633.36	12900.76	12961.76	12707.10	0.88	0.05	0.41	95.47 (0.01)
7 Profile	−6174.40	12488.80	12795.64	12865.64	12573.41	0.90	0.04	0.36	162.57 (0.01)
8 Profile	−6154.81	12467.62	12813.91	12892.91	12563.11	0.90	0.03	0.37	39.18 (0.01)
9 Profile	−6120.08	12416.17	12801.92	12889.92	12522.54	0.88	0.03	0.36	69.45 (0.01)

Values in bold indicate the selected optimal model

Abbreviations: Akaike Information Criterion (AIC), Bayesian Information Criterion (BIC), Consistent Akaike Information Criterion (CAIC), Sample-Size Adjusted BIC (SABIC), Bootstrap Likelihood Ratio Test (BLRT)

**Fig. 1:**
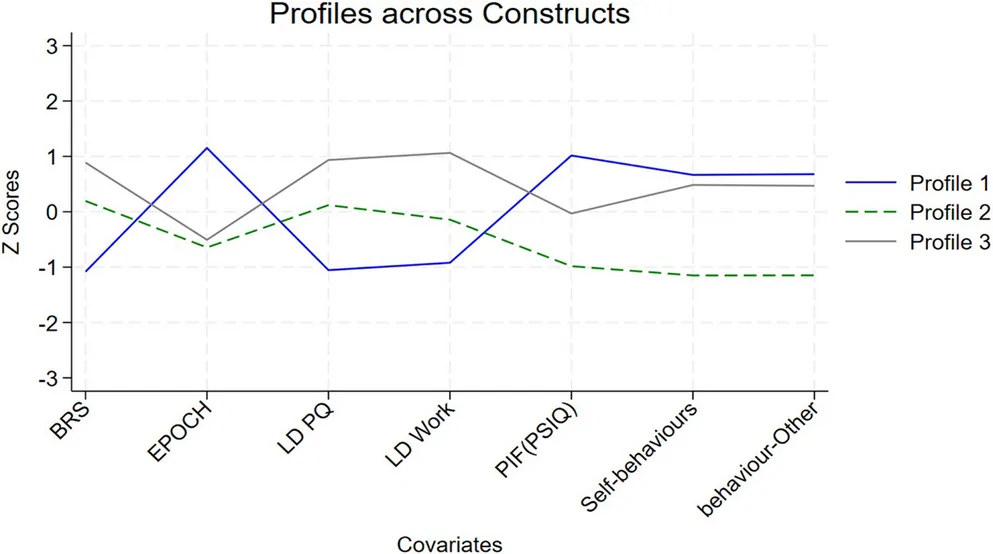
Line*-*graph showing the relationship between the study variable and latent profiles

Table 3 presents the estimated means for the constructs of PIF, Professionalism, Leadership, Resilience, and Well-being by profiles. A three-profile latent model was identified with distinct patterns across constructs. Profile 1 (*Conscientious Followers*) was characterized by higher scores on PIF (transitioning from student to qualified doctor), well-being, and professionalism, but lower scores on resilience and leadership. Profile 2 (*Development Group*) demonstrated the lowest scores across leadership, professionalism, Professional Identity Formation, and well-being, while showing moderate levels of resilience. Profile 3 (*Resilient Leaders*) was characterized by lower scores on well-being, moderate levels of Professional Identity Formation and professionalism, and higher scores on resilience and leadership. Additionally, Fig. 1 shows standardized (z-score) profiles across these constructs. Positive values indicated above-average levels, while negative values indicated below-average levels.

**Table 3:** Estimated latent profile means for professional identity formation, professionalism, leadership, resilience and well-being indicators among medical students

		Profiles		
Covariates		1	2	3
PIF-(PISQ) (First day student doctor- qualified doctor)		2.93	2.30	2.60
Professionalism (Self-behaviours)		50.76	28.94	48.58
Professionalism (Behaviour-others)		11.20	6.40	10.65
Leadership Personal qualities (LD PQ)		1.36	1.72	1.97
Leadership work with others (LD Work)		1.26	1.57	2.05
Resilience (BRS)		1.98	2.33	2.52
Well-being (EPOCH)		3.41	3.28	3.29

Abbreviations: BRS – Brief Resilience Scale; EPOCH – Engagement, Perseverance, Optimism, Connectedness, Happiness; LD- Leadership; PIF – Professional Identity Formation; PISQ- Professional Self-Identity Questionnaire

Based on the latent profile analysis, we further examined factors associated with the identified profiles. Univariate analyses (Appendix 1) indicated that several demographic variables differed significantly across the three profiles (*p* < 0.05). Variables found to be significant in the univariate analysis were subsequently included as independent predictors in the multivariate analysis. The students’ study program was also included as a covariate to ensure the robustness of the results and to control for its potential influence. Table 4 presents the findings of the multinomial logistic regression, comparing membership in the Development Group and Resilient Leaders profiles relative to the reference group, Conscientious Followers. Results indicated significant associations between several demographic factors and latent profile membership. Specifically, nationality emerged as a significant predictor for both the Development Group and Resilient Leaders profiles. Middle Eastern students were significantly more likely to belong to the Resilient Leaders profile. Year of study was also significantly associated with profile membership, students in Year 3 and beyond were more likely to be classified as Resilient Leaders compared to Conscientious Followers, suggesting a potential developmental trend in leadership and resilience as students advance through their academic journey.

**Table 4:** Multivariate level: multinomial logistic regression results for latent profile membership and covariates

Profiles: Conscientious followers (Ref.)	Development group			Resilient leaders		
Covariates	Odds-Ratio (OR)	*P*-Value	95% CI	Odds-Ratio (OR)	*P*-Value	95% CI
Student Program						
DEM (Ref.)	1.000	--	--	1.000	--	--
GEM	0.545	0.091	0.27, 1.10	1.626	0.082	0.94, 2.81
Gender						
Female *(Ref.)*	1.000	--	--		--	--
Male	1.761	0.054	0.99, 3.14	1.349	0.170	0.88, 2.07
Nationality						
Irish *(Ref.)*	1.000	--	--	1.000	--	--
Middle Eastern	0.216	0.031	0.21, 0.93	2.161	0.008	1.22, 3.83
North American	0.554	0.128	0.26, 1.18	0.767	0.399	0.41, 1.42
Others	0.617	0.213	0.29, 1.32	0.876	0.696	0.45, 1.70
Year of Study						
Year 1 *(Ref.)*	1.000	--	--	1.000	--	--
Year 2	1.697	0.284	0.64, 4.47	1.359	0.375	0.69, 2.68
Year 3	2.345	0.103	0.84, 6.52	2.198	0.038	1.04, 4.64
SC1	2.718	0.028	1.11, 6.65	2.541	0.003	1.37, 4.71
SC2	3.014	0.012	1.27, 7.15	1.557	0.171	0.83, 2.93

(Ref.) =reference category, CI=confidence interval

## Discussion

This study has shown for the first-time evidence that PIF, professionalism, leadership, resilience, and well-being form distinct subgroups in medical students. In addressing our research question, we identified three distinct profiles, *Conscientious Followers*,* Development Group and Resilient Leaders*, which reveal meaningful differences in how students experience and demonstrate various aspects of their professional growth. These results demonstrate that professional identity formation is not the same for all students, instead, it develops in different ways depending on individual characteristics and experiences [4, 13]. The identification of these latent profiles offers novel insights into the heterogeneous nature of PIF and these related constructs, providing a conceptual lens to guide future theory-driven research on professional development in medical education.

Although the present study identified distinct profiles of PIF, professionalism, leadership, resilience, and well-being, the cross-sectional design does not permit conclusions regarding whether these profiles reflect characteristics students brought with them to medical school, characteristics shaped by the educational environment, or an interaction between the two. Nevertheless, recent longitudinal findings from the PILLAR study demonstrated that Professional Identity Formation increased significantly throughout medical training, whereas professionalism, leadership, and resilience did not show comparable developmental change [14]. These findings suggest that some aspects of professional development may not develop automatically during medical training and may require deliberate educational support. Consequently, the profiles identified in the present study should be viewed as reflecting heterogeneity in students’ professional development and may provide a useful framework for future research examining how curricular, personal, and contextual factors influence profile membership and development over time.

Profile 1 (*Conscientious Followers*) was characterized by higher scores on Professional Identity Formation (transitioning from student to qualified doctor), psychological well-being, and professionalism, but lower scores on resilience and leadership. This pattern reflects students who have strong professional identity and well-being, but may still struggle with leadership and coping under stress. While their professional development and adjustment appear positive, low resilience and leadership could limit their ability to navigate challenges or take on leadership roles (41). Research suggests that resilience and leadership are critical factors for sustaining long-term success and adaptability in high-pressure environments [42]. Students in this profile, although exhibiting positive professional development, may require additional support to fully capitalize on their potential. Targeted interventions, on resilience training, leadership development, and structured mentorship, could help this group strengthen adaptive skills while maintaining their professional growth [43].

Profile 2 (*Development Group*) demonstrated the lowest scores across leadership, professionalism, PIF, and well-being, while showing moderate levels of resilience. This pattern suggests a cohort of students who may be in a state of professional stagnation: they possess some identification with the profession, but their professional behaviours are still in need of development, and they may struggle to translate their emerging identity into confident, competent action. Previous research suggest that these difficulties may arise from limited self-efficacy, suboptimal clinical exposure, or a lack of structured support systems [44, 45], although this cannot be explored in the present data. This aligns with prior literature indicating development of a robust professional identity requires both internal motivation and reinforcement through authentic role modelling and consistent constructive feedback [12, 13]. Students in this profile may be particularly vulnerable to disengagement, stress, and uncertainty, which can hinder their ability to integrate professional values into practice. To address this, more developmental and foundational interventions should be considered. Medical educators could implement structured clinical placements with strong supervision, guided opportunities to practice professional behaviours, targeted interventions to build confidence in their abilities, and consistent ongoing feedback. These strategies can help bridge the gap between identity awareness and behavioural expression, promoting both psychosocial adaptation and professional maturity [46, 47].

Profile 3 (*Resilient Leaders*), comprised students who, despite having lower scores on well-being and moderate levels of PIF, had higher scores on resilience, leadership and professionalism. This pattern indicates strong adaptive and leadership capacities, although the transition toward a more consolidated and operationalized professional identity remains incomplete. These findings are consistent with developmental models of professional identity formation, which emphasize the need for integration of internalized values with social and clinical competence as students move from early-stage learners to fully functioning professionals [13]. Students within this group may be especially well-suited for near-peer mentoring or leadership development initiatives, which could simultaneously reinforce their emerging identity and support the professional growth of their peers [48, 49]. Notably, recent evidence from a cross-sectional study at RCSI indicates that resilience is negatively correlated with mental health difficulties, including depression, anxiety, and burnout, and remains stable across demographic and educational variables [50]. This suggests that the high resilience observed in Profile 3 students may serve as a protective factor, enabling them to maintain leadership and professional performance even when well-being is comparatively lower. Therefore, resilience appears to support adaptive functioning and professional development in medical students, bridging the gap between current challenges and long-term identity consolidation.

The multinomial logistic regression results revealed that nationality and year of study were significant predictors of latent profile membership. Specifically, Middle Eastern students were more likely to belong to the Resilient Leaders profile, suggesting potential cultural or contextual influences on resilience and leadership development. These results are consistent with prior research indicating that cultural background influences coping strategies, interpersonal dynamics and leadership perceptions in medical education contexts [51, 52]. For instance, research shows that in collectivist cultural contexts, protective factors for academic resilience tend to operate more effectively through group-based supports and adaptability [53]. Importantly, these findings should be interpreted within the context of a programmatic assessment framework delivered within a single institution. RCSI educates a highly diverse international student population. Consequently, students from different cultural and national backgrounds are exposed to a shared curriculum, assessment system, and educational environment. The observed differences in profile membership therefore should not be interpreted as reflecting inherent characteristics of particular demographic groups, but rather as potentially reflecting complex interactions between cultural background, prior educational experiences, individual characteristics, and the shared institutional context [14]. Additionally, students in higher academic years were more likely to belong to the Resilient Leaders profile, suggesting a developmental progression in leadership and professional identity as students advance through their training. This is consistent with longitudinal evidence showing that medical students’ professional understandings and role-maturation evolve over time [54]. Increased exposure to clinical environments and observation of role models likely foster leadership and resilient professional identity development, as students engage with real-world challenges and modelling behaviour [55]. Although gender was not a significant predictor in the present analysis, demographic and educational factors may nevertheless influence students’ professional development experiences and warrant further investigation in future research. As medical schools increasingly educate diverse student populations, educators should consider how factors such as nationality, stage of training, and local educational context may influence the expression and development of these or similar constructs when interpreting and applying these findings.

From an educational perspective, these findings reinforce the importance of moving beyond a “one-size-fits-all” approach to professional development in medical education. The identification of distinct learner profiles suggests that students may benefit from different forms of support depending on their developmental needs [12, 13]. For example, students with lower resilience and leadership scores may benefit from targeted coaching, mentorship, and leadership development opportunities, whereas students demonstrating strong resilience and leadership may benefit from near-peer mentoring or advanced leadership roles [48, 49]. As medical schools increasingly adopt Professional Identity Development curricula, person-centred approaches may help educators identify areas of strength and vulnerability within student populations and inform the design of more responsive educational supports [12, 13]. Future research should examine whether tailored educational strategies can influence profile membership and support positive professional development trajectories over time.

### Strengths and Limitations

The strength of this study is in its innovative assessment of co-development of PIF together with professionalism, leadership, resilience, and well-being among medical students. Additionally, the employment of well-validated instruments strengthens the reliability and validity of the results. Our sample included students from a variety of backgrounds, which adds value to our findings and supports their relevance in diverse educational settings. There are some limitations that need consideration. A potential limitation of this analysis is the decision to merge DEM and GEM cohorts. While this approach increased the overall sample size and statistical power, the two groups may differ in important demographic characteristics such as age and nationality (with GEM students more likely to be older and of Irish nationality). These differences could potentially influence the observed outcomes. The exclusive reliance on self-reported data introduces the possibility of social desirability and recall biases, which may affect the accuracy of responses. Additionally, profile subgroup assignments are inherently probabilistic, as a result, individual classifications represent estimates rather than definitive categorizations, with potential for misclassification [17]. Importantly, the cross-sectional quantitative design limits our ability to make causal conclusions and prevents us from understanding how these constructs may evolve in students over time. Furthermore, the study design does not permit determination of whether the identified latent profiles reflect characteristics that students brought with them to medical school e.g., personality characteristics shaped by the educational environment or how these interact. Consequently, caution is warranted when interpreting profile membership as a product of educational experiences alone. Longitudinal research is needed to examine the stability of these profiles over time and to better understand the relative contributions of personal, contextual, and curricular influences to professional development. Additionally, replication of these profiles is required to be more confident of their robustness. Future work could address these limitations, for example, by linking PILLAR data to summative assessment scores to examine related outcomes. Additionally, adopting a mixed-methods approach that integrates qualitative insights with quantitative profiles would provide a deeper understanding of how students’ experiences shape their professional growth and the evolution of these profiles over time. 

## Conclusion

This study provides important evidence into the complexity of professional identity formation among medical students by identifying distinct subgroups with varying levels of identity integration, professionalism, leadership, resilience, and well-being. These findings highlight the potential value of person-centred approaches for understanding heterogeneity in professional development among medical students and suggest that future research should explore whether tailored, developmentally appropriate interventions can better support students at different stages of their identity formation journey.

## Supplementary Information

Below is the link to the electronic supplementary material.

Supplementary Material 1 (PDF 323 KB)

Supplementary Material 2 (DOCX 33.5 KB)

## Data Availability

The data supporting the findings of this study may be obtained from the corresponding author upon reasonable request and approval from the RCSI Research Ethics Committee. Requests for access should be directed to dicknsimbe24@rcsi.ie.

## Appendix 1

**Table:** Univariate analysis of Latent Profile Membership and Covariates

Profiles: Conscientious followers (Ref.)	Development Group			Resilient leaders		
*Covariates*	*Odds-Ratio (OR)*	*P-Value*	*95% CI*	*Odds-Ratio (OR)*	*P-Value*	*95% CI*
Student Program						
DEM (Ref.)	1.000	--	--	1.000	--	--
GEM	0.695	0.225	0.38, 1.25	1.038	0.861	0.68, 1.57
Gender						
Female *(Ref.)*	1.000	--	--	1.000	--	--
Male	1.356	0.245	0.81, 2.27	1.588	**0.021**	1.07, 2.35
Nationality						
Irish *(Ref.)*	1.000	--	--	1.000	--	--
Middle Eastern	0.629	0.172	0.32, 1.22	1.909	**0.011**	1.16, 3.15
North American	0.561	0.124	0.26, 1.17	0.918	0.779	0.50, 1.67
Others	0.696	0.315	0.34, 1.41	0.773	0.426	0.41, 1.46
Age						
Year of Study						
Year 1 *(Ref.)*	1.000	--	--	1.000	--	--
Year 2	1.668	0.296	0.64, 4.36	1.329	0.403	0.68, 2.59
Year 3	2.585	0.062	0.95, 7.01	1.969	0.061	0.99, 4.01
SC1	2.625	**0.033**	1.08, 6.37	1.389	**0.005**	1.31, 4.37
SC2	2.939	**0.014**	1.25, 6.92	1.558	0.163	0.84, 2.91

*(Ref.)* reference category, *CI* confidence interval
