# Supplementary material for: Latent Profiles of Professional Identity Formation, Professionalism, Leadership, Resilience, and Well-being among Medical Students
Source: Med Sci Educ. 2026 Jul 16;36(5):2219–29. doi: 10.1007/s40670-026-02820-6 (PMC13723466; doi:10.1007/s40670-026-02820-6)
Supplement: Supplementary file 1 [file 40670_2026_2820_MOESM1_ESM.pdf]

Latent Profiles of Professional Identity Formation, Professionalism, leadership, Resilience, and Well-being Among Medical Students

Dick Nsimbe, MSc[1], Anne Hickey, PhD[1], Denis W. Harkin, MD, PhD[2], Abdelsalam Bensaaud, PhD[2], Áine Ryan, PhD[2], Frank Doyle, PhD[1]

[1]Department of Health Psychology, School of Population Health, RCSI University of Medicine and Health Sciences, Dublin, Ireland

[2]Centre for Professionalism in Medicine and Health Sciences at RCSI University of Medicine and Health Sciences, Dublin, Ireland

Comment Number	Comment	Response	Page Number
**Reviewer 1**	**Feedback on the introduction/background/rationale/theoretical framework:** *Solid framework.*<br>**For Research Articles: Feedback on the design, methods and statistics:** *Design and methods are good.*<br>**Feedback on the results/discussion or main body of the manuscript:** *Data is presented clearly.*<br>**Feedback on the conclusions/summary:** *Conclusion is clearly worded and based on the data presented.*<br>**Feedback on the conclusions/summary:** *Conclusion is clearly worded and based on the data presented.*<br>**Feedback on the appropriateness and timeliness of the reference list:** *Broad sample of references which are relevant to the topics. The older references are relevant as a foundation for the newer developments*.<br>**Feedback on the tables and figures:** *Tables and figures are clear and relevant.*	Reviewer 1 was positive about the manuscript and did not request any changes. We thank the reviewer for their time and positive comments.	
**Reviewer 2**	**Feedback on the introduction/background/rationale/theoretical framework:**<br>This is a very well written manuscript and I appreciated the effort that clearly went into putting it together. I was also very interested in the idea of identifying groups of students in the way that you did and that you considered trying to identify and characterize these groups. That being said, I have some very minor suggestions to request/suggest:	Thank you for your positive comment.	
**Abstract**	Very well written - no changes requested.	Thank you for your comment	
**Introduction**	Page 2, Lines 49 - 51: rewrite the sentence to remove the double negative. It will flow better. The final paragraph of the introduction would benefit from a research question to really set up exactly what you are doing.	Thank you for these helpful suggestions. We revised the sentence to improve clarity and readability.<br>**Original:** *"Ryan et al. (5,10) provided empirical evidence that*	Page 3 & 4

		these constructs are not independent, but instead contribute collectively to professional development and identity formation."<br>**Revised**: *"Ryan et al. (5,11) provided empirical evidence that these constructs are interrelated and contribute collectively to professional development and identity formation."*<br><br>In addition, we incorporated a research question into the final paragraph of the Introduction to more clearly frame the study aims and rationale, as follows:<br>*"Accordingly, this study sought to answer the following research question: Can distinct latent profiles of medical students be identified based on PIF, professionalism, leadership, resilience, and their relationship to well-being, and what demographic factors are associated with membership in these profiles?"*	
**Methods**	In the methods, its typically expected that you report you received research approval to be able to use the data/survey the students? Please include this as a small ethics section. If you did not need to acquire approvals, please state that instead.	We thank the reviewer for this suggestion. Ethical approval was reported in both the Methods and Declarations sections of the original manuscript. To improve clarity, we expanded the statement in the Methods section to report adherence to STROBE guidelines, ethical approval from the RCSI Research Ethics Committee (REC202005016), and the informed consent procedures followed in the study, as follows:<br>*"This study was reported in accordance with the Strengthening the Reporting of Observational Studies in Epidemiology (STROBE) guidelines (26). The completed STROBE checklist is provided in the Supplementary Material. Ethical approval for the study was granted by the RCSI Research Ethics Committee (REC202005016), and all participants provided informed consent prior to participation."*	Page 4

**Discussion**	Your manuscript would benefit from more development of the limitations section which is rather buried by the strengths of the study	Thank you for this suggestion. We have expanded the limitations section to provide a more balanced discussion of the study's constraints. In particular, we added the following:<br><br>*"Importantly, the cross-sectional quantitative design limits our ability to make causal conclusions and prevents us from understanding how these constructs may evolve in students over time. Furthermore, the study design does not permit determination of whether the identified latent profiles reflect characteristics that students brought with them to medical school e.g., personality characteristics shaped by the educational environment or how these interact. Consequently, caution is warranted when interpreting profile membership as a product of educational experiences alone. Longitudinal research is needed to examine the stability of these profiles over time and to better understand the relative contributions of personal, contextual, and curricular influences to professional development. Additionally, replication of these profiles is required to be more confident of their robustness"*	Page 13
**Reviewer 3**	**Feedback on the introduction/background/rationale/theoretical framework:**<br>Thank you for sharing your work. As someone who coaches learners on professional and communication issues, I recognize the importance of professional identity formation and the need for schools to support our learners' development. I appreciate that your manuscript emphasizes the importance and calls on medical educators to recognize that there is no "one size fits all" approach. As you refine this paper for this journal, I encourage you to discuss more the application and the next steps your findings suggest so that those less interested in the method by which you found these profiles can move them to the next step.	Thank you, we agree that the educational implications and practical applications of the identified profiles could be more articulated. In response, we have expanded the Discussion, as follows:<br>*"From an educational perspective, these findings reinforce the importance of moving beyond a "one-size-fits-all" approach to professional development in medical education. The identification of distinct learner profiles suggests that students may benefit from different forms of support depending on their*	Page 12-13

		developmental needs (12,13). For example, students with lower resilience and leadership scores may benefit from targeted coaching, mentorship, and leadership development opportunities, whereas students demonstrating strong resilience and leadership may benefit from near-peer mentoring or advanced leadership roles (49,50). As medical schools increasingly adopt Professional Identity Development curricula, person-centred approaches may help educators identify areas of strength and vulnerability within student populations and inform the design of more responsive educational supports (12,13). Future research should examine whether tailored educational strategies can influence profile membership and support positive professional development trajectories over time."	
**Other comments**	-Please include the full name in the first reference to LPA and consider offering a clearer explanation as to why this type of analysis matters to med science educators. In this case, I think it is less about the value of the method generally and more about what the "person-centered" approach means and brings.	Thank you for the suggestion. We have revised the first mention of LPA to provide the full term, Latent Profile Analysis, and expanded the accompanying text to clarify the educational relevance of a person-centred approach, as follows: *"Applying a person-centred approach such as Latent Profile Analysis (LPA) allows for the identification of latent subgroups of students whose profiles across these competencies may reflect distinct pathways of professional identity formation. For medical educators, this provides an opportunity to better understand variation in learners' developmental experiences and support needs rather than assuming a uniform pattern of professional development across all students."*	Page 3
	-The well-being construct is occasionally omitted from the larger groups of concepts (e.g., p2, lines 55-60 defines the other terms, but not well-being). Given that you mentioned using a scale to measure well-being, it would be helpful for the reader to have that term defined as well.	Thank you, we agree that the role of well-being was not sufficiently articulated in the original manuscript. Conceptually, well-being is considered an outcome of multiple interacting factors, rather than an inherent	Page 3 and 5

	-Additionally, paragraphs 1 and 3 on page 2 also leaves out well-being. Have scholars not identified it as being interrelated with professionalism, leadership, and resilience (page 2, line 4, 8), or is there another reason for its absence?<br><br>-The Methods section noted that the PILLAR method included four instruments and, again, leaves off well-being. Can you elaborate on why you chose to put it into your study? Doing so will help the reader better understand it's importance and difference from resilience. After writing this, I went and read the PILLAR study and saw that they combined well-being and resilience. That information seems useful to include here as well.	component of professional identity formation, which contributed to its less treatment in the original Background. However, we agree that its role required clearer explanation and have revised the manuscript in three ways.<br><br>First, we added a definition of well-being in the opening paragraph of the Background, as follows:<br><br>*"…and well-being reflects positive psychological functioning and flourishing, including engagement, perseverance, optimism, connectedness, and happiness, which support students' ability to thrive during medical training (10)."*<br><br>Second, we revised the conceptual rationale for integrating well-being as an outcome in the PILLAR methodology paper. We now state: *"Previous research has shown that professionalism, leadership, and resilience are important contributors to the professional development and identity formation of medical students (5,11). Although previous studies have traditionally examined these constructs in isolation (3,7,9), emerging evidence suggests they are conceptually linked and tend to co-occur within individuals (5,12,13). Ryan et al. (5,11) provided empirical evidence that these constructs are interrelated and contribute collectively to professional development and identity formation. Although well-being is conceptually viewed as an outcome arising from multiple interacting personal and educational factors, rather than an inherent component of professional identity formation itself, the original PILLAR methodology incorporated well-being alongside resilience as an important indicator of students'*	

		psychosocial functioning and capacity to flourish during medical training."<br><br>Third, we expanded the Methods (Variables) section to explain why well-being was included in the present study and to clarify its distinction from resilience. We now state:<br>*"The PILLAR assessment included well-established and standardized instruments designed to evaluate PIF, professionalism, leadership, resilience, and well-being (11). Consistent with the original PILLAR methodology, well-being was included alongside the other constructs because positive psychological functioning is considered an important component of students' professional development and capacity to thrive during medical training. Although conceptually distinct from professional identity formation, well-being provides an important indicator of how students experience and adapt to the demands of medical training. While resilience reflects the ability to recover from stress and adversity, well-being captures broader dimensions of flourishing, including engagement, perseverance, optimism, connectedness, and happiness.*"	
	**For Research Articles: Feedback on the design, methods, and statistics:**<br>*-I read this article as more a methods paper than a results-oriented paper. Is it possible to reduce the length of the methods/results section (or move it to an appendix) so more time could be used explaining the three profiles?*	Thank you for this suggestion. We agree that, for a medical education audience, the educational interpretation and practical implications of the identified profiles are of primary importance. We retained the methodological detail necessary to ensure transparent reporting in accordance with the STROBE guidelines and to facilitate reproducibility of the Latent Profile Analysis, we revised the manuscript to place greater emphasis on the educational interpretation of the three profiles and their relevance to medical education. These	Page 4-7

		revisions have been incorporated throughout the manuscript, with the specific changes detailed in the relevant responses above and below.	
	**Feedback on the results/discussion or main body of the manuscript:**<br>*-After reading the results, I immediately wondered how race/class/gender may have affected the results. I saw you addressed it in the last paragraph of the discussion. Could you expand more on that so those who will use your results can tailor it more to their demographics*	Thank you, we agree that the potential influence of demographic and contextual factors warranted further discussion. In response, we expanded the Discussion, as follows:<br><br>*"…Importantly, these findings should be interpreted within the context of a programmatic assessment framework delivered within a single institution. RCSI educates a highly diverse international student population. Consequently, students from different cultural and national backgrounds are exposed to a shared curriculum, assessment system, and educational environment. The observed differences in profile membership therefore should not be interpreted as reflecting inherent characteristics of particular demographic groups, but rather as potentially reflecting complex interactions between cultural background, prior educational experiences, individual characteristics, and the shared institutional context (14)."*<br><br>*"…Although gender was not a significant predictor in the present analysis, demographic and educational factors may nevertheless influence students' professional development experiences and warrant further investigation in future research. As medical schools increasingly educate diverse student populations, educators should consider how factors such as nationality, stage of training, and local educational context may influence the expression and development of these or similar constructs when interpreting and applying these findings."*	Page 12

	**Feedback on the conclusions/summary:**<br>*-I didn't get a good enough sense of the profiles and what the medical schools did/did not do to foster those profiles. It was unclear to me if these were personality traits the individuals brought to medical school or developed there. Thus, I was hesitant to accept the final claims that this study provided evidence of the value of person centered approaches or that tailored, developmentally appropriate interventions would make a difference in these areas.*	We agree that the cross-sectional design of the present study does not permit conclusions regarding whether the identified profiles reflect characteristics students brought with them to medical school, characteristics shaped by the educational environment, or an interaction between the two. To address this concern, we have expanded the Discussion, as follow:<br><br>*"Although the present study identified distinct profiles of PIF, professionalism, leadership, resilience, and well-being, the cross-sectional design does not permit conclusions regarding whether these profiles reflect characteristics students brought with them to medical school, characteristics shaped by the educational environment, or an interaction between the two. Nevertheless, recent longitudinal findings from the PILLAR study demonstrated that Professional Identity Formation increased significantly throughout medical training, whereas professionalism, leadership, and resilience did not show comparable developmental change (14). These findings suggest that some aspects of professional development may not develop automatically during medical training and may require deliberate educational support. Consequently, the profiles identified in the present study should be viewed as reflecting heterogeneity in students' professional development and may provide a useful framework for future research examining how curricular, personal, and contextual factors influence profile membership and development over time."*<br><br>We have also moderated our conclusions as follows, in line with the reviewer's concerns:<br>*"This study provides important evidence into the complexity of*	Page 10-11 and 13

		professional identity formation among medical students by identifying distinct subgroups with varying levels of identity integration, professionalism, leadership, resilience, and well-being. These findings highlight the potential value of person-centred approaches for understanding heterogeneity in professional development among medical students and suggest that future research should explore whether tailored, developmentally appropriate interventions can better support students at different stages of their identity formation journey."	
	**Feedback on the appropriateness and timeliness of the reference list:**<br>-the presence of several statistical and quantitative studies suggests to me that this may be better placed in a journal with methods focus.	Thank you for your comment, while the study employs quantitative and person-centred analytical methods, our primary objective is not methodological development but rather to advance understanding of professional identity formation, professionalism, leadership, resilience, and well-being in medical education. The statistical methods were selected as appropriate tools to address an educational research question concerning heterogeneity in students' professional, and is not written as a tutorial paper. We therefore believe that the manuscript aligns well with the scope of Medical Science Educator, given its focus on educational theory, learner development, and implications for curriculum design and student support.	
	**Feedback on the tables and figures:**<br>-I do not have the training to full appreciate or comment on these	Thank you for this comment. This is noted. No specific concerns or suggested revisions were raised.	
