# Supplementary material for: Latent Profiles of Professional Identity Formation, Professionalism, Leadership, Resilience, and Well-being among Medical Students
Source: Med Sci Educ. 2026 Jul 16;36(5):2219–29. doi: 10.1007/s40670-026-02820-6 (PMC13723466; doi:10.1007/s40670-026-02820-6)
Supplement: Supplementary file 2 [file 40670_2026_2820_MOESM2_ESM.docx]

**STROBE Statement—Checklist of items that should be included in reports of *cohort studies***

|  | Item No | Recommendation | **Page No./Section** |
| --- | --- | --- | --- |
| **Title and abstract** | 1 | (*a*) Indicate the study’s design with a commonly used term in the title or the abstract | Title (p. 1); Abstract (p. 2) |
|  |  | (*b*) Provide in the abstract an informative and balanced summary of what was done and what was found | Abstract (p. 2) |
| Introduction | | |  |
| Background/rationale | 2 | Explain the scientific background and rationale for the investigation being reported | Background (pp. 2–4) |
| Objectives | 3 | State specific objectives, including any prespecified hypotheses | Background, final paragraph with research question (pp. 4) |
| Methods | | |  |
| Study design | 4 | Present key elements of study design early in the paper | Methods: Study design (pp. 4-5) |
| Setting | 5 | Describe the setting, locations, and relevant dates, including periods of recruitment, exposure, follow-up, and data collection | Methods: Setting and Participants; Study Sample (pp. 4-5) |
| Participants | 6 | (*a*) Give the eligibility criteria, and the sources and methods of selection of participants. Describe methods of follow-up | Methods: Setting and Participants; Study Sample (pp. 4-6) |
|  |  | (*b*) For matched studies, give matching criteria and number of exposed and unexposed | Not applicable — this was not a matched study. |
| Variables | 7 | Clearly define all outcomes, exposures, predictors, potential confounders, and effect modifiers. Give diagnostic criteria, if applicable | Methods: Professional Identity Formation; Professionalism; Leadership; Resilience and Well-Being (pp. 4–7 |
| Data sources/ measurement | 8* | For each variable of interest, give sources of data and details of methods of assessment (measurement). Describe comparability of assessment methods if there is more than one group | Methods: Professional Identity Formation; Professionalism; Leadership; Resilience and Well-Being; Data Sources (pp. 4–7) |
| Bias | 9 | Describe any efforts to address potential sources of bias | Methods: Statistical Analysis (pp. 7–8); Discussion: Strengths and Limitations (pp. 13) |
| Study size | 10 | Explain how the study size was arrived at | Methods: Study Sample (p. 5) |
| Quantitative variables | 11 | Explain how quantitative variables were handled in the analyses. If applicable, describe which groupings were chosen and why | Methods: Statistical Analysis (pp. 7-8) |
| Statistical methods | 12 | (*a*) Describe all statistical methods, including those used to control for confounding | Methods: Statistical Analysis (pp. 7-8) |
|  |  | (*b*) Describe any methods used to examine subgroups and interactions | Methods: Statistical Analysis (pp. 7-8); No formal interaction terms were tested. |
|  |  | (*c*) Explain how missing data were addressed | Methods: Statistical Analysis (pp. 7-8) |
|  |  | (*d*) If applicable, explain how loss to follow-up was addressed | Not Applicable |
|  |  | (*e*) Describe any sensitivity analyses | Not applicable — sensitivity analyses were not conducted. |
| Results | | |  |
| Participants | 13* | (a) Report numbers of individuals at each stage of study—eg numbers potentially eligible, examined for eligibility, confirmed eligible, included in the study, completing follow-up, and analysed | Methods: Data Sources (pp. 6–7); Study Size (p. 5); Results: Participant Characteristics, Table 1 (p. 8). Final analysed sample reported as N = 679. Numbers at all recruitment stages were not explicitly reported. |
|  |  | (b) Give reasons for non-participation at each stage | Not explicitly reported. |
|  |  | (c) Consider use of a flow diagram | Not included. |
| Descriptive data | 14* | (a) Give characteristics of study participants (eg demographic, clinical, social) and information on exposures and potential confounders | Results: participant characteristics and demographics in Table 1 (p. 8) |
|  |  | (b) Indicate number of participants with missing data for each variable of interest | Not explicitly reported in the manuscript. |
|  |  | (c) Summarise follow-up time (eg, average and total amount) | Not applicable |
| Outcome data | 15* | Report numbers of outcome events or summary measures over time | Results: Tables 2–4 and Figure 1 (pp. 8–10), reporting latent profile model fit indices, profile-specific summary measures, and associations between participant characteristics and profile membership. |
| Main results | 16 | (*a*) Give unadjusted estimates and, if applicable, confounder-adjusted estimates and their precision (eg, 95% confidence interval). Make clear which confounders were adjusted for and why they were included | Results: Unadjusted estimates reported in Appendix 1; adjusted multinomial logistic regression estimates (ORs, 95% CIs, p-values) reported in Table 4 (p. 10). Covariates adjusted for included programme, gender, nationality, and year of study. |
|  |  | (*b*) Report category boundaries when continuous variables were categorized | Not applicable |
|  |  | (*c*) If relevant, consider translating estimates of relative risk into absolute risk for a meaningful time period | Not applicable — this study did not estimate relative risks. |
| Other analyses | 17 | Report other analyses done—eg analyses of subgroups and interactions, and sensitivity analyses | Results: Tables 2–4 (pp. 8–10). Model fit comparisons, univariate analyses, and multinomial logistic regression analyses of profile membership were conducted. No interaction or sensitivity analyses were reported. |
| Discussion | | |  |
| Key results | 18 | Summarise key results with reference to study objectives | Discussion: Key findings summarized in relation to the study objective of identifying latent profiles and their demographic correlates (pp. 10–12). |
| Limitations | 19 | Discuss limitations of the study, taking into account sources of potential bias or imprecision. Discuss both direction and magnitude of any potential bias | Discussion: Strengths and Limitations (pp. 13–14). |
| Interpretation | 20 | Give a cautious overall interpretation of results considering objectives, limitations, multiplicity of analyses, results from similar studies, and other relevant evidence | Discussion (pp. 10–13). |
| Generalisability | 21 | Discuss the generalisability (external validity) of the study results | Discussion (pp. 12–13). |
| Other information | | |  |
| Funding | 22 | Give the source of funding and the role of the funders for the present study and, if applicable, for the original study on which the present article is based | Funding/Support (p. 14) |

*Give information separately for exposed and unexposed groups.

**Note:** An Explanation and Elaboration article discusses each checklist item and gives methodological background and published examples of transparent reporting. The STROBE checklist is best used in conjunction with this article (freely available on the Web sites of PLoS Medicine at http://www.plosmedicine.org/, Annals of Internal Medicine at http://www.annals.org/, and Epidemiology at http://www.epidem.com/). Information on the STROBE Initiative is available at http://www.strobe-statement.org.
